# A 77-GHz Six-Port Sensor for Accurate Near-Field Displacement and Doppler Measurements

**DOI:** 10.3390/s18082565

**Published:** 2018-08-06

**Authors:** Homa Arab, Steven Dufour, Emilia Moldovan, Cevdet Akyel, Serioja O. Tatu

**Affiliations:** 1 Institut National de la Recherche Scientifique—Centre Énergie Matériaux Télécommunications, Montréal, QC H5A 1K6, Canada; moldovan@emt.inrs.ca (E.M.); serioja.tatu@emt.inrs.ca (S.O.T.); 2 École Polytechnique de Montréal, Montréal, QC H3C 3A7, Canada; steven.dufour@polymtl.ca (S.D.); cevdet.akyel@polymtl.ca (C.A.)

**Keywords:** antenna array, coupler, Doppler radar, millimetre wave, passive component, reflection coefficient, sensor, six-port interferometer

## Abstract

A continuous-wave (CW) radar sensor design based on a millimetre-wave six-port interferometer is proposed. A complete sensor prototype is conceived of, fabricated and measured at 77 GHz for short-range professional and industrial applications. This sensor is designed to measure distances and Doppler frequencies with high accuracy, at a reasonable cost. Accurate phase measurements are also performed using the six-port technology, which makes it a promising candidate for CW radar sensing applications. Advances in the performance and functionality of six-port sensors are surveyed to highlight recent progress in this area. These include improvements in design, low power consumption, high signal to noise ratio, compactness, robustness and simplicity in realization. Given the fact that they are easy to fabricate, due to the lack of active circuits and being highly accurate, it is expected that six-port sensors will significantly contribute to the development of human tracking devices and industrial sensors in the near future. The entire circuit prototype, including the transmitter, the receiver antenna, the six-port interferometer and the four power detectors have been integrated on a die. The circuit is fabricated using a hybrid integrated technology on a 127-μm ceramic substrate with a relative permittivity of $\varepsilon_{r}=9.8$. Calibrated tuning forks are used to assess the performance of the six-port sensor experimentally for various frequencies.

## 1. Introduction

Direction finding and ranging techniques are attracting wide interest for their use in various industrial applications. Designed and manufactured radar sensors are usually based on laser interferometry or on cameras, which have high costs and cannot easily penetrate through dirt, steam or fog. They can therefore only be used in limited situations. However, in harsh industrial environments, or for high precision measurements, new approaches must be found, using update rates in the kHz domain.

With the growing interest in radars for short-range applications, several mm-wave sensor technologies have been studied and tested in recent years [1,2,3,4,5]. Operating in the mm-wave band using small low-cost components make it possible to use a large bandwidth, therefore leading to high range resolution and accuracy.

Radar sensor systems consist of a combination of two main parts: the radar hardware and the digital signal processing (DSP) unit. The radar hardware is composed of the transmitter (TX) and receiver (RX) radio frequency (RF) components, antennas, power detectors, amplifiers, etc.

Various radar technologies such as continuous-wave (CW), frequency-modulated continuous-wave (FMCW) and pulsed-Doppler radars are used in numerous applications [6,7,8]. The first Doppler sensor was designed in the early 1970s using heavy and expensive waveguide components at the Western Electric Company. mm-wave technology makes it possible to integrate several radar hardware architectures on a small chip. Single-chip Doppler radars based on a bipolar transistor direct-conversion quadrature radar receiver, on a digital IF receiver (DRX), on a hybrid CW-FMCW radar and on a six-port radar are examples of the hardware architectures used [1,9,10,11,12,13,14,15].

On the signal processing side, techniques such as small-angle approximations, complex demodulation, arctangent demodulation, adaptive DC calibration, noise cancellation, distortion cancellation and I/Q mismatch mitigation have been developed [9,10,16,17,18]. Each technique leads to a design that is appropriate for a given application, each with its benefits and drawbacks.

Given this framework, the entire mm-wave RF front-end and the T/R modules of the proposed short-range radar are designed, fabricated and measured over a bandwidth of 70–82 GHz. The system is based on a six-port interferometer, and an I/Q demodulation technique is used to recover the desired information. The sensor can send and process all these signals. However, the CW radar is chosen due to its simplicity and range accuracy [19].

The six-port circuit, introduced by F. Engen, has been known since the 1960s [20]. It has been used, for the last 30 years, as a two-port vector network analyser (VNA) [21]. The six-port theory has recently made its way into other fields such as for material characterization, receivers and radars [22,23,24,25,26,27]. The six-port interferometer can be implemented using various architectures consisting of hybrid couplers, power dividers and phase shifters [28,29]. In this paper, a six-port interferometer is designed, experimentally characterized and validated for mm-wave signal quadrature down-conversion. Miniaturized hybrid microwave integrated circuit (MHMIC) technology is used in the fabrication process.

To validate the measurement accuracy of the proposed sensor, the reflected wave phase shift is used to measure the mechanical oscillation frequency of a target. A single-tone unmodulated CW signal transmits to the target, and a non-linear modulated phase reflects to the radar receiver. In what follows, an outline of the proposed CW sensor design and its limitations will be given based on system simulations and a measurement setup using a 77-GHz wideband six-port receiver system and a two-patch T/R antenna array.

## 2. Theory and Basic Equations of CW Radar

**Figure 1:**
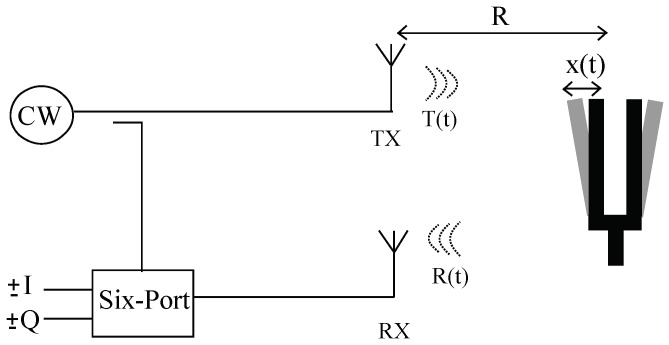
Simplified block diagram of the CW Doppler sensor.

The Doppler CW sensor transmits an unmodulated RF signal to the target, which reflects part of the signal back to the receiver of the sensor. The moving target phase modulates the carrier RF signal in a non-linear way. The basic structure of the CW sensor is illustrated in Figure 1.

The signal transmitted by the waveform generator is given by:

(1) $$T\left(t\right)=A_{t}\cos\left(2\pi ft+\theta (t)\right),$$

where *f* is the frequency of the transmitted signal, θ(t) is the phase noise from the RF generator and $A_{t}$ is the amplitude of the transmitted signal. The received signal can then be approximated by:

(2) $$R\left(t\right)=A_{r}\cos\left(2\pi ft-\frac{4\pi (R+x(t))}{\lambda }+\theta \left(t-\frac{2R}{c}\right)\right),$$

where $A_{r}$ is the amplitude of the receiver signal, *R* is the range of the target, x(t) is the position of the target, λ is the wavelength of the carrier and *c* is the speed of light. As illustrated in Figure 1, the transmitted signal is used as a local oscillator (LO) signal to down-convert the received signal as:

(3) $$B\left(t\right)=A_{b}\cos\left(\frac{4\pi (R+x(t))}{\lambda }+\theta \left(t\right)-\theta \left(t-\frac{2R}{c}\right)\right).$$

The residual phase noise, θ(t)−θ(t−2R/c), is negligible due to the coherent nature of the sensor. In fact, both the TX and the LO signals have the same source and phase noise, which can cancel each other, for example in the case of short-range sensor applications [9]. Equation (3) shows that the vibration of the target (x(t)) modulated the phase of the received signal. A special technique is then required to recover accurate information about the position of the target.

In this project, tuning forks are used as targets, which can produce exponentially-damped sinusoidal movements. Therefore, to simplify the analysis, and without any loss of generality, we consider the sinusoidal movement to be in the steady-state response of the fork movement. As discussed in [17,30], the baseband signal at the output of the sensor can be expressed as a Bessel function expansion of order *n*, $J_{n}\left(x\right)$. For a single tone target movement, x(t)=msin(2πft), the baseband output signal can be expressed as:

(4) $$B\left(t\right)=\sum_{n=-\infty }^{\infty }J_{n}\left(\frac{4\pi m}{\lambda }\right)\cos\left(2\pi nf\right)\cos\left(\frac{4\pi R}{\lambda }\right).$$

Equation (4) shows that harmonics will be created at the baseband output signal due to the non-linear property of the cosine transfer function. The movement frequency is obtained from the fundamental frequency of B(t), and the amplitude of the vibrations of the fork, and the residual phase, can accurately be determined from the rate of the harmonics. The harmonics generated by the non-linear phase modulation can be used to recover the desired information about movement of the target [9,18].

## 3. Radar Sensor Components

This section describes each module of the proposed CW radar. The detailed block diagram of each module, with measurement and simulation results, is given.

### 3.1. The Six-Port Interferometer

**Figure 2:**
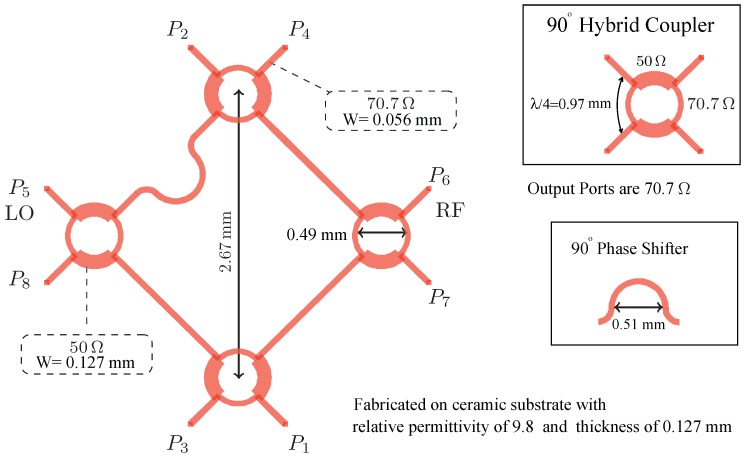
Layout of the six-port interferometer. LO, local oscillator.

**Figure 3:**
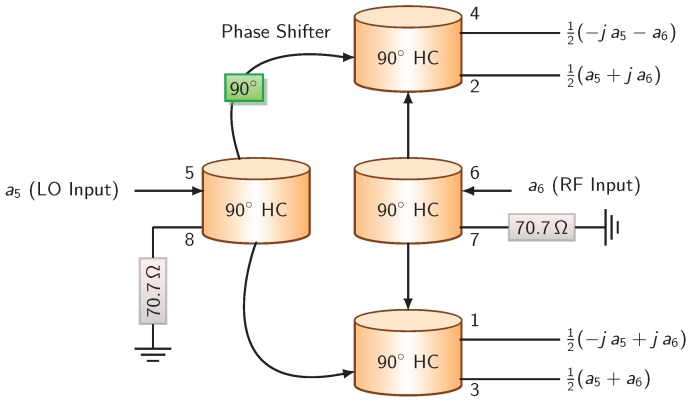
Block diagram of the six-port interferometer.

The proposed six-port interferometer is a linear passive mm-wave circuit, which is the combination of four 90 hybrid couplers and of a 90 phase shifter implemented by a microstrip transmission line. The six-port layout and its block diagram are illustrated in Figure 2 and Figure 3. The physical dimensions and characteristic impedances are given in Figure 2.

The six-port circuit under study receives two input signals at the RF and LO ports (the received and reference signals). Ports 1–4 are connected to four power detectors to measure the phase and amplitude differences between two input signals. Ports 7 and 8 are connected to matched loads. For an ideal six-port phase correlator, it can be shown that:

(5) $$\left[\begin{array}{c} b_{1}\\ b_{2}\\ b_{3}\\ b_{4}\\ b_{5}\\ b_{6} \end{array}\right]=\left[\begin{array}{cccccc} 0 & 0 & 0 & 0 & -j & +j\\ 0 & 0 & 0 & 0 & +1 & +j\\ 0 & 0 & 0 & 0 & +1 & +1\\ 0 & 0 & 0 & 0 & -j & -1\\ -j & +1 & +1 & -j & 0 & 0\\ +j & +j & +1 & -1 & 0 & 0 \end{array}\right]\left[\begin{array}{c} a_{1}\\ a_{2}\\ a_{3}\\ a_{4}\\ a_{5}\\ a_{6} \end{array}\right],$$

where $b_{1}$, $b_{2}$, $b_{3}$ and $b_{4}$ represent the output waves at Ports 1–4, $a_{5}$ is the input reference signal and $a_{6}$ is the received signal reflected from the target. We note that $a_{1}$ to $a_{4}$ are equal to zero for matched circuits connected to the six-port outputs. Given the amplitude ratio α of the six-port interferometer, input signals for varying frequencies and phases can be expressed as:

(6) a5=αaexpjωt+ϕ5(t);a6=αaexpjωt+ϕ6(t).

The normalized output power can be expressed as a function of the normalized input powers by:

(7) b1=12(−ja5+ja6);b2=12(−ja5+ja6);b3=12(−ja5+ja6);b4=12(−ja5−ja6).

Four diode-based power detectors are connected to the output ports of the circuit, and two differential amplifiers can be used in order to obtain the in-phase output and the quadrature signals (I and Q). These I and Q signals, and the related complex demodulated Γ, are expressed as: [31]:

(8) $$\begin{array}{c} I\left(t\right)=V_{3}\left(t\right)-V_{1}\left(t\right)=\alpha \;a^{2}\;k\cos\left[\Delta \omega (t)+\Delta \phi (t)\right];\\ Q\left(t\right)=V_{4}\left(t\right)-V_{2}\left(t\right)=\alpha \;a^{2}\;k\sin\left[\Delta \omega (t)+\Delta \phi (t)\right];\\ \Gamma (t)=I(t)+j\;Q(t), \end{array}$$

where the constant *k* is related to the gain of the differential amplifiers and to the efficiency of the power detectors. Using these baseband voltage values, we can calculate the instantaneous frequency, the phase and the amplitude differences between the received and the reference signals.

**Figure 4:**
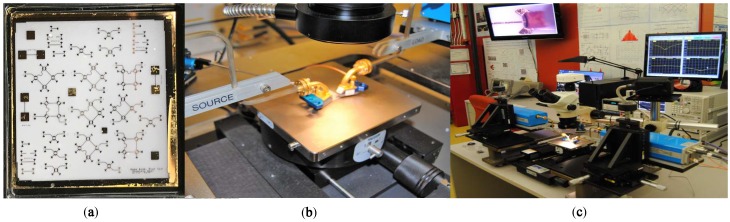
(**a**) The coplanar probe with a pitch of 150 μm. (**b**) The fabricated circuit for full-port characterization. (**c**) Two-port mm-wave measurement setup.

As shown in Figure 4a, various circuit configurations are fabricated on a ceramic substrate in order to perform two-port measurements. The measurement results will be used to validate the six-port circuit design and to generate a multi-port circuit model (based on multiple two-port measurements) to be used in advanced system simulations. The measurement setup includes a vector network analyser (E 8362 PNA, Keysight, Santa Rosa, CA, USA), a mm-wave head controller (N 5260A, Keysight, Santa Rosa, CA, USA), two mm-wave extenders to cover 60–90 GHz (OML S12MS-A, Keysight, Santa Rosa, CA, USA) and two bended WR-12 waveguides used to connect the coplanar probes with a pitch of 150 μm (cf. Figure 4b,c).

**Figure 5:**
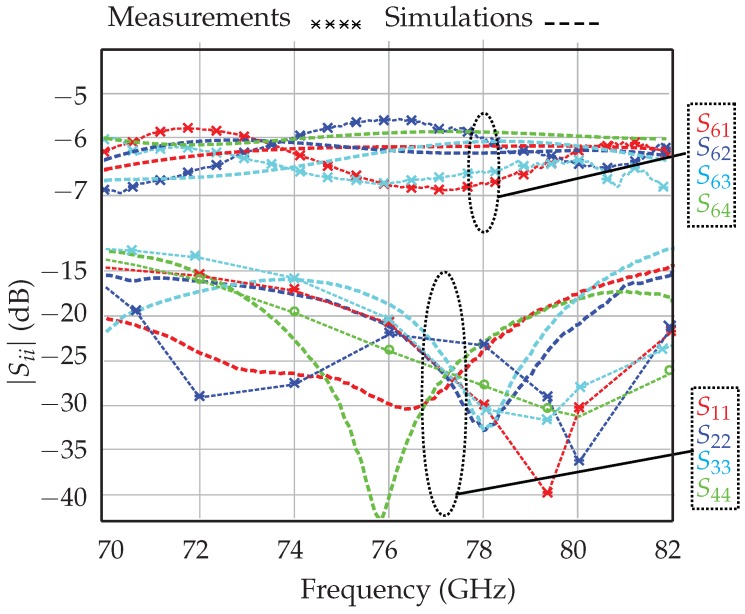
Return losses at the four output ports and the transmission between the RF port ($S_{6i}$) and the four output ports.

In order to perform fast and more accurate measurements, through-reflect-line (TRL) calibration kits are fabricated in the same substrate. Since the probe tips are coplanar waveguides, transitions from the microstrip to the coplanar waveguides are required. Furthermore, in order to avoid via holes and to ensure measurement repeatability, quarter-wavelength open lines and butterfly wing open-circuited sectors are used as mm-wave short circuits [32]. The reflection coefficients at the detector ports ($S_{11}$–$S_{44}$) and the transmission between the input RF port ($S_{6}$) and the output ports are shown in Figure 5 (more details can be found in [33,34,35]).

**Figure 6:**
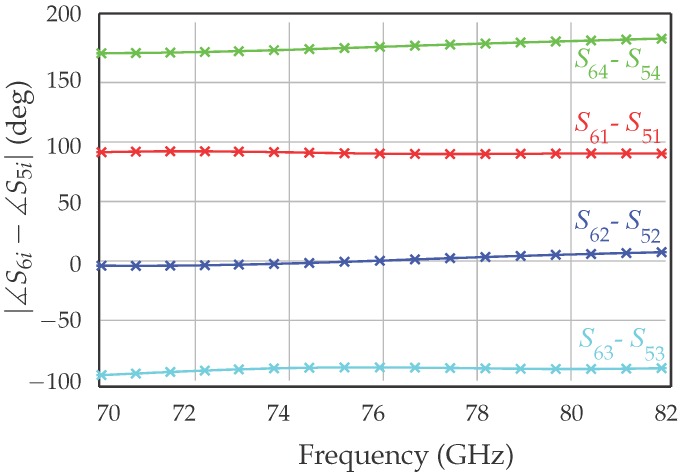
Measured transmission phase between the output ports.

These results prove that the output ports are well matched over a wide frequency band. Moreover, the transmission between the input RF port and the four output ports is close to the theoretical value of −6 dB over the same 12-GHz band. The phase shift of 90 between the output ports is illustrated in Figure 6.

Measurement results show a maximum error of ±2.5% at 77 GHz and less than ±5% over the 12-GHz bandwidth.

To validate the operation of the six-port interferometer model, obtained with VNA measurements, a harmonic balance simulation is used. The measurement-based model is used with the Advanced Design System (ADS) software simulator by Keysight Technologies. The phase difference between the LO and the RF inputs is swept over 360. The expected voltage values are:

(9) $$\begin{array}{c} V_{3,1}=\frac{k}{2}\;a^{2}\left(1\pm \cos(\Delta \phi )\right);\\ V_{4,2}=\frac{k}{2}\;a^{2}\left(1\pm \sin(\Delta \phi )\right). \end{array}$$

**Figure 7:**
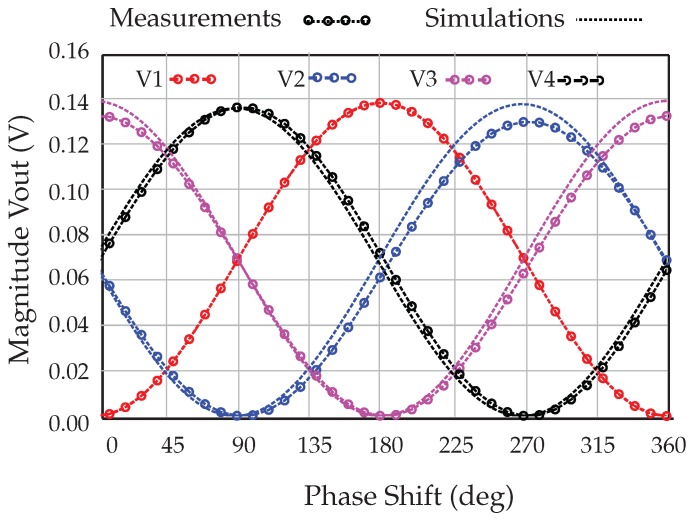
Magnitude of the power detected voltages at 77 GHz.

The measurement and simulation results are shown in Figure 7. The six-port interferometer exhibits quasi-sinusoidal output voltage signals with a phase difference of 90, with similar amplitudes.

### 3.2. Power Detector

**Figure 8:**
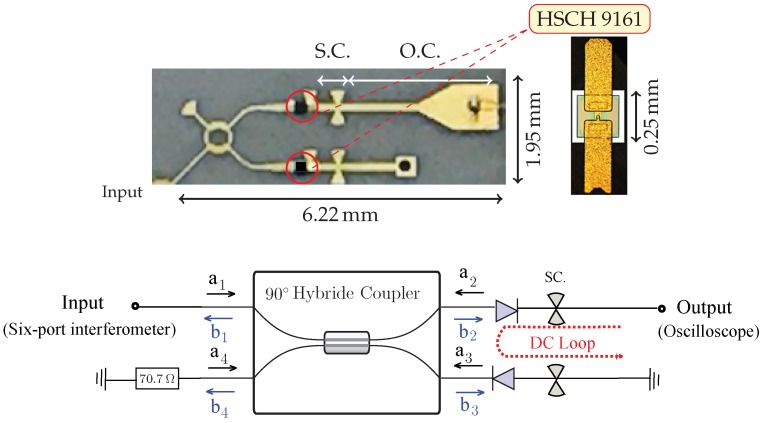
Picture of the fabricated miniaturized hybrid microwave integrated circuit (MHMIC) diode-based power detector and a schematic diagram of the power detector.

A good six-port sensor design must allow the accurate measurement of high-frequency power ratios. The four outputs of each six-port circuit are usually connected to power detectors that deliver a voltage related in a linear way to the power of the RF signal at the four output ports. Figure 8 shows the details of the fabricated power detector using two Schottky diodes (HSCH 9161) and a hybrid coupler, as a way to improve input matching [36]. Four power detectors of this type are used in the sensor to extract magnitude and phase information about the reflected signal from the target.

Based on the diagram of Figure 8 and using two identical diodes at ports 2 and 3 ($\Gamma =a_{2}/b_{2}=a_{3}/b_{3}$), we have that:

(10) $$\left[\begin{array}{c} b_{1}\\ b_{2}\\ b_{3}\\ b_{4} \end{array}\right]=\left[\begin{array}{cccc} 0 & j & 1 & 0\\ j & 0 & 0 & 1\\ 1 & 0 & 0 & j\\ 0 & 1 & j & 0 \end{array}\right]\left[\begin{array}{c} a_{1}\\ \Gamma \;b_{2}\\ \Gamma \;b_{3}\\ 0 \end{array}\right].$$

Equation (10) shows that the reflection coefficient at the input port is zero,

(11) $$b_{1}=\frac{\Gamma }{\sqrt{2}}\left(jb_{2}+b_{3}\right)=0,$$

and the input power of each diode is a function of the input power of the power detector ($a_{1}$) and of the reflection coefficient of the diode (Γ),

(12) $$P_{D}=|b_{2}{|}^{2}-|a_{2}{|}^{2}=(1-{\left|\Gamma \right|}^{2})\frac{1}{2}{\left|a_{1}\right|}^{2}.$$

For the design of the power detector, 70.7 Ω microstrip terminations and resistor test kits were used to verify the required value of 100 Ω per square for the integrated loads. In addition, mm-wave RF short circuits are implemented with quarter wavelength sectors, avoiding many via-holes.

The simulated return losses of the diode-based power detector are given in Table 1. However, the designed power detectors provide more than a 10-GHz bandwidth, which is sufficient not only for the designed CW radar sensor, but also for high resolution FMCW radar applications. In fact, the bandwidth of the proposed diode-based power detector is wider than the available bandwidth at 77 GHz (75.5–81 GHz). The conversion losses are around 10 dBm in the input power range of 5–−50 dBm. The four outputs are usually transformed into in-phase (I) and quadrature (Q) components of a down-converted signal using differential amplifiers.

### 3.3. WR-12 Rectangular Waveguide to Microstrip Line Transition

**Figure 9:**
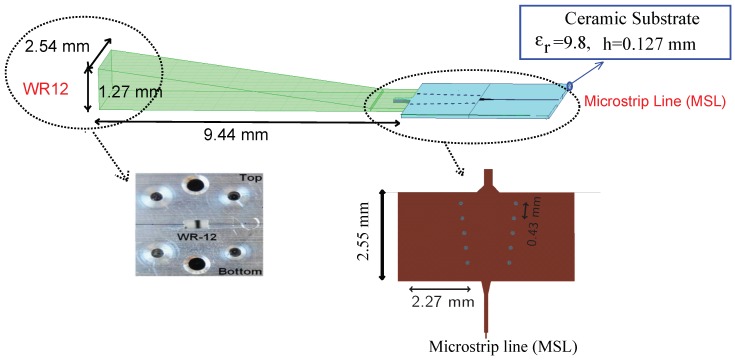
WR-12 rectangular waveguide to microstrip line transition.

A waveguide to microstrip transition requires a low transmission loss, a low voltage standing wave ratio (VSWR), a low insertion loss, enough bandwidth and a structure that is as simple as possible and easy to set up. Based on these guidelines, an E-plane coupling probe and a substrate integrated waveguide tapered transition structure are used for the standard rectangular waveguide (WR-12 which is designed for 60–90 GHz) to microstrip line (MSL) transition. In the proposed transition, an impedance matching and an EM field mode transformation between the WR-12 and the MSL are required. Impedance matching is required from the higher characteristic impedance of the WR-12 to 70.7 Ω of the MSL. On the other hand, it is needed to transit from the fundamental TE10 mode of the rectangular waveguide to the microstrip line quasi-TEM mode. For these aims, a microstrip wave adapter and a tapered integrated waveguide transformer are used. Impedance matching is achieved by varying the widths of the transmission lines in microstrip wave adapter. The waveguide (SIW) is used in the transition to connect and match the waveguide part to the microstrip line. The metallized via-holes of the SIW allow reducing unwanted substrate waves and shielding the transition from backside radiation leakage. The mechanism of the electromagnetic field matching for this arrangement has been detailed in [37]. The transition is fabricated in a very thin high-permittivity ceramic substrate ($\varepsilon_{r}=9.8$, h=127μm) as the other parts of the sensor radar. The layout and all dimensions are illustrated in Figure 9.

**Figure 10:**
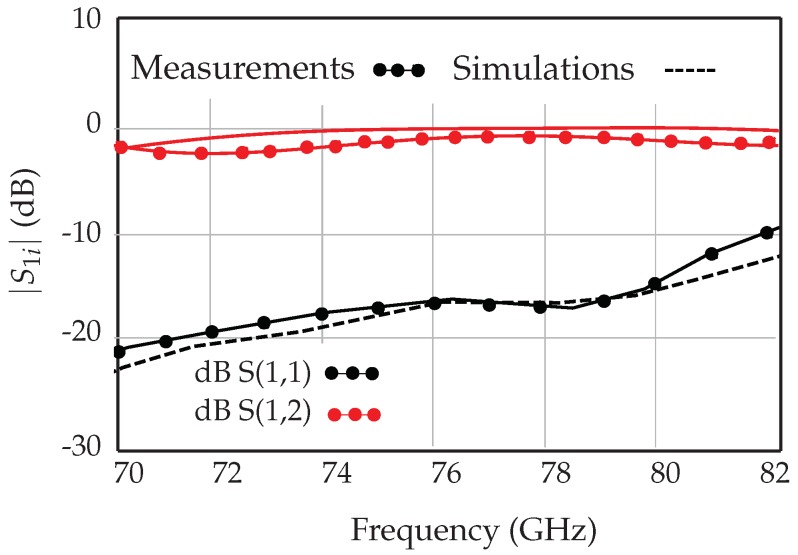
WR-12 waveguide to microstrip line transition.

The measured and simulated insertion loss and the return loss of the fabricated transition prototype are shown in Figure 10. The return loss is less than −10 dB in the entire bandwidth of 70–82 GHz, showing a good agreement between the MSL and the WR-12 waveguide. The insertion loss of each MSL to WR-12 transition is less than 2.5 dB over the same bandwidth. The measurement and simulation results show that the proposed WR-12 waveguide to microstrip line transition meets the RF front-end sensor requirements.

### 3.4. Antenna

**Figure 11:**
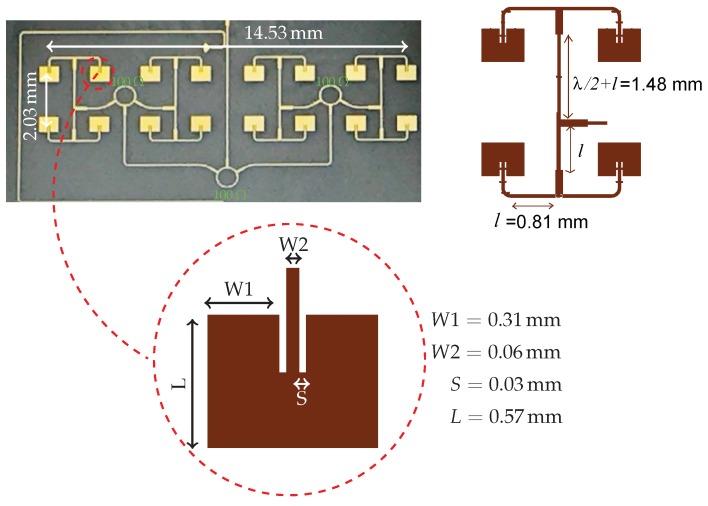
Picture of the fabricated 8×2 antenna array and the ADS layout with dimensions of the antenna element and 2×2 antenna array.

A 16-dBi gain 77-GHz mm-wave 16×1 microstrip patch antenna array is designed and fabricated. In order to reduce the size of the entire prototype, and to facilitate its integration with other integrated passive devices, the proposed antenna array has been designed on the same ceramic die. The performance of the antenna array is investigated based on bandwidth, gain and radiation efficiency. It is directly connected to the six-port circuit in the transmitter and the receiver part. A picture of the fabricated MHMIC antenna array and the dimensions of the antenna elements and array are illustrated in Figure 11.

The antenna array structure consists of 16 patch elements and a parallel feed network composed of three rounded shape Wilkinson power dividers, along with the 70.7 Ω transmission lines. The Wilkinson power divider was selected as having the ideal three-port network properties including lossless, reciprocal and matching at all ports, at millimetre-wave frequencies [38].

**Figure 12:**
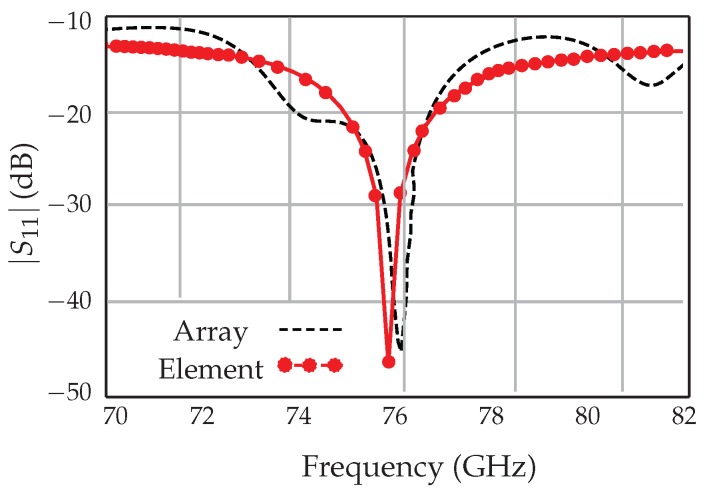
Simulated reflection coefficient of the 77–GHz antenna element and array.

**Figure 13:**
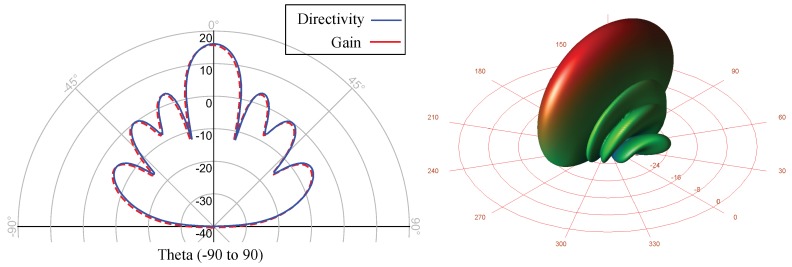
Simulated 3D radiation pattern of the antenna array at 77 GHz.

The antenna operating at 77 GHz is designed and optimized using the ADS simulation software. The simulated scattering parameters of the antenna array and the antenna element are given in Figure 12. Input reflection of less than −10 dB is achieved for both the antenna element and the array over a wide bandwidth range of 70–82 GHz. The simulated 3D radiation pattern of the antenna array at 77 GHz is shown in Figure 13. The use of two lines of eight patches produces a directional beam that is optimal for measurements in a perpendicular direction to the line of patches (aligned in 0–180 in the figure).

The simulated peak gains at 77 GHz are 6.3 dBi for the element and 16.2 dBi for the array. The proposed antenna and array design are promising for mm-wave applications due to its wide bandwidth, low cost, high efficiency and low profile. This antenna is included in the transmitter and receiver sections, in the same substrate of the six-port interferometer and all designed radar sensor components. They are completely integrated into a 16.83 mm × 13.94 mm ceramic substrate. This fabrication process reduces the size and the price of the circuit, with good performance.

## 4. Prototype and Test Results

**Figure 14:**
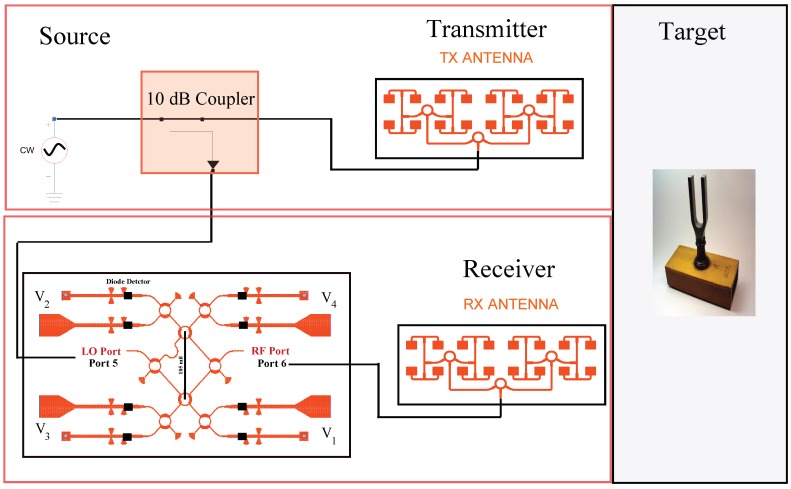
Block diagram of the 77-GHz sensor.

For performance measurements, a sensor prototype was realized and several simulations and measurements were made. A 77–GHz CW signal having a 0-dBm output power is transferred to the transmitter antenna, and the reference signal is derived through a 10-dB parallel line coupler from a CW source. The coupler is chosen in order to keep the diode voltages in a square low region with enough power for the diodes. To achieve this, the power of the local oscillator has to be between −10 and −30 dBm. Considering a 10-dB parallel line coupler and the transition line losses between the coupler and the six-port circuit, the input power at the local port is around −15 dBm. The passive six-port circuit and diode detectors will interferometrically generate I and Q signals. The frequency and the phase of the derived quadrature signals represent the phase difference between the transmitter and the receiver signals. From the I and Q signals, the movement of the target and its range from the sensor can be accurately calculated [39,40]. The block diagram of the 77-GHz sensor is illustrated in Figure 14.

### 4.1. Simulation Results in ADS

To validate the proposed schematic, the measurement results of the six-port circuit and the parameters of the designed antenna are imported into the ADS simulator. The ration of received power to transmitted power can be simulated based on the radar equation as:

(13) $$\begin{array}{c} P_{r}=P_{t}G_{t}G_{r}\frac{\lambda^{2}}{{\left(4\pi \right)}^{2}R^{2}}\sigma \frac{4\pi }{\lambda^{2}}\frac{\lambda^{2}}{{\left(4\pi \right)}^{2}R^{2}}, \end{array}$$

where σ represents the radar cross-section (RCS), $G_{r}$ is the receiver gain, $G_{t}$ is the transmitter gain, $P_{r}$ is the received power and $P_{t}$ is the transmitted power. This equation is for a far-field target where the RCS is significantly smaller than the range. For a short-range sensor, the near-field RCS of the target (a metallic plate), $4\pi w^{2}h^{2}/\lambda^{2}$, is considered [41]. Using this equation for modelling the target using the ADS simulator gives good results when compared to theoretical values and to measurement results. Furthermore, the received signal power is reduced by 12 dB as the range between the sensor and the target doubles. In order to have a strong enough signal at the baseband output, a low-noise amplifier is useful for medium to long-range sensor applications.

**Figure 15:**
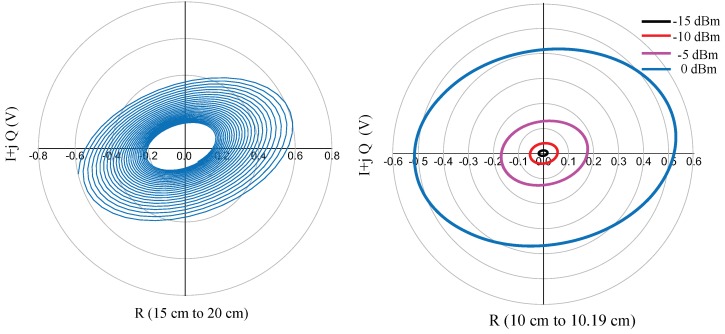
Simulation results for the displacement of the target near the antenna for various input powers.

In the first simulation, the target, a metallic plate with a cross-section of 0.01 m${}^{2}$, is moved continuously with a velocity of 5 m/s over a displacement range of 15–20 cm. Amplifiers with a 20-dB gain are used at the two IF output ports of the circuit. This creates a spiral-shaped complex representation of the displacement near the antenna, which is illustrated in Figure 15. The I/Q signals for four different input power levels from 0 dBm–−15 dBm, and a displacement of 1.95 mm (λ/2) are also illustrated in Figure 15. These results show that when the I and Q signals are larger than the noise level, the input power level cannot be less than −10 dBm.

In a second simulation, the target was placed at a fixed distance and was moved continuously with a range of λ/2, corresponding to a 360 phase shift of the reflected wave from the target. The simulation results show a closed loop, which should ideally be a circle according to the theory of six-port interferometers. The distortion is related to the use of raw six-port output signals, without any calibration. The accuracy of the sensor is not affected since frequency measurements do not depend on amplitude distortions. It also shows that this architecture is well suited for short-range distance sensing.

It is important to mention that range measurement can be incorrect due to the uncertainty related to the obtained phase shift Δϕ of 2π. An accurate position can only be detected within the range of λ/2. A target with a well-defined movement and range is needed for validating measurement results with the results obtained through simulations. Tuning forks with four different frequencies are therefore used with the ADS simulations and measurement test bench.

The forks are located at a distance of R=25 cm from the sensor. The small oscillation of the forks is modelled in the ADS simulator. For modelling the target, a phase modulator is excited using a voltage source having a frequency equal to the fork oscillation frequency. A phase shifter is used to simulate the mechanical vibrations within a known range of ±λ/4, based on:

(14) $$\Delta \phi =\frac{4\pi \Delta R}{\lambda }.$$

**Figure 16:**
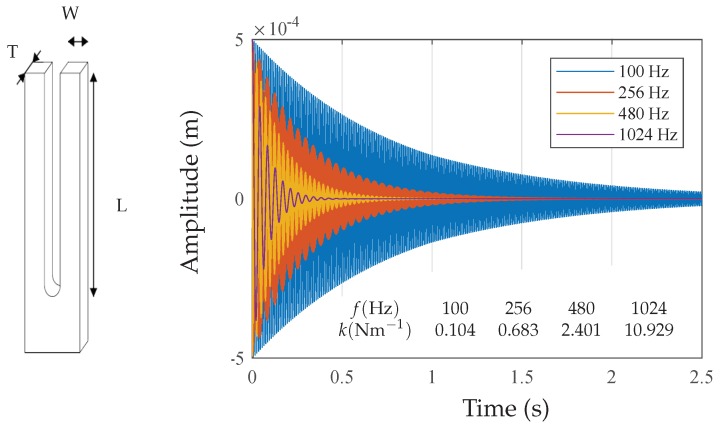
Oscillation of the tuning forks for various frequencies and the associated *k*.

The vibration of the fork is modelled using [42]:

(15) $$m\;\frac{d^{2}x}{dt^{2}}+\gamma \;m\;\frac{dx}{dt}+k\;x=F,$$

where *m* represents the mass, γ is a damping coefficient, *k* is the spring constant and *F* is the amplitude of the driving force. The constant *k* depends on the geometry and the material of the fork ($k=\frac{1}{4}ET{\left(w/L\right)}^{3}$ in our case). The constant *k* for the four selected frequencies is given in Figure 16.

**Figure 17:**
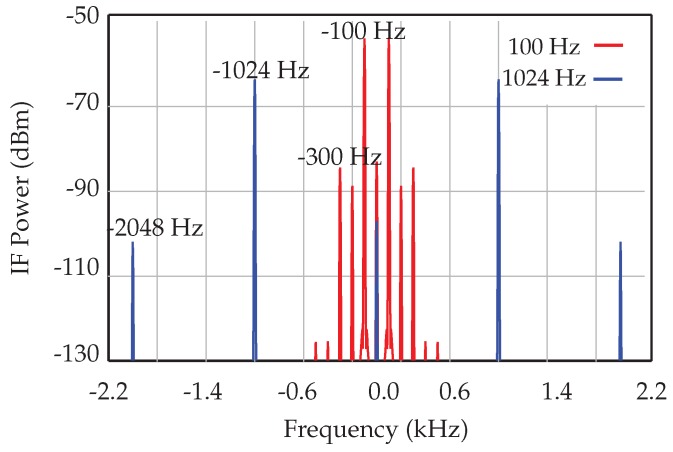
Simulation results for the spectra of the IF signal for the 100-Hz and 1024-Hz tuning forks.

As shown in Figure 16, the transient solution for the fork vibrations is an exponentially-damped sinusoidal function. However, if the fork is steadily driven sinusoidally by the force Fcos(ωt), then the solution of Equation (18) becomes a sinusoidal function:

(16) $$\begin{array}{c} x(t)=B\sin(\omega t);\\ B\left(\omega \right)=Q\frac{F}{k}\frac{\omega_{0}}{\omega }/\sqrt{1+Q^{2}{\left(\frac{\omega_{0}}{\omega }-\frac{\omega }{\omega_{0}}\right)}^{2}};\\ \tan\left(\theta \right)={\left[Q(\frac{\omega_{0}}{\omega }-\frac{\omega }{\omega_{0}})\right]}^{-1}, \end{array}$$

where *Q* and $\omega_{0}$ are defined with respect to *m*, γ and *k* ($\omega_{0}^{2}=k/m$ and $Q/\omega_{0}=m/\gamma$). As mentioned earlier, this non-linear phase modulation will generate a harmonic on the baseband signal. Figure 17 shows the spectra of the IF signal for two different fork frequencies (100 Hz and 1024 Hz).

The produced harmonics on the baseband signal can be expanded as a Bessel function of the first kind:

(17) B(t)=cos[4πRλ+4πBsin(ωt)λ];B(t)=Re[exp(j4πBsin(ωt)λ)+exp(4πRjλ)].

The exponential sinusoidal term can be extended using a Fourier series as:

(18) Re[exp(j4πBsin(ωt)λ)]=∑n=−∞∞Jn4πBλexpjnωt.

A linear approximation of the Bessel function can be used since the amplitude of the tuning forks’ vibrations is small (4πB/λ≪1). We then have:

(19) $$J_{n}\left(\frac{4\pi B}{\lambda }\right)=\frac{1}{2n!}{\left(\frac{4\pi B}{\lambda }\right)}^{n}.$$

**Figure 18:**
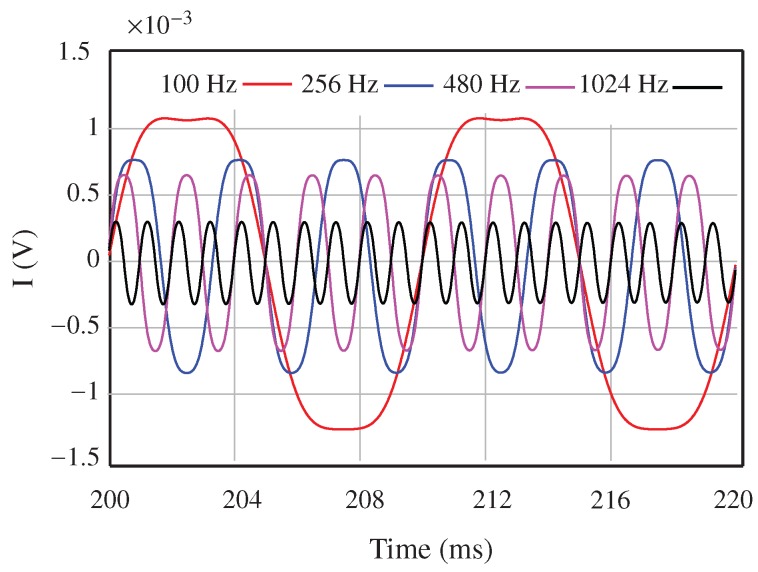
Magnitude of the IF signals (in-phase (I)).

These parameters of the mechanical vibrations of the target are modelled in the ADS simulator by using a phase modulator. The amplitude of the movement is less than λ/8, which creates a phase shift smaller than 90. The simulation results for the I signal are shown in Figure 18. The results for the Q signal are the same with a 90 phase shift. It can be seen that the simulated results match very well with the theoretical analysis, and the vibrations of the forks are modelled accurately.

### 4.2. System Measurement Results

**Figure 19:**
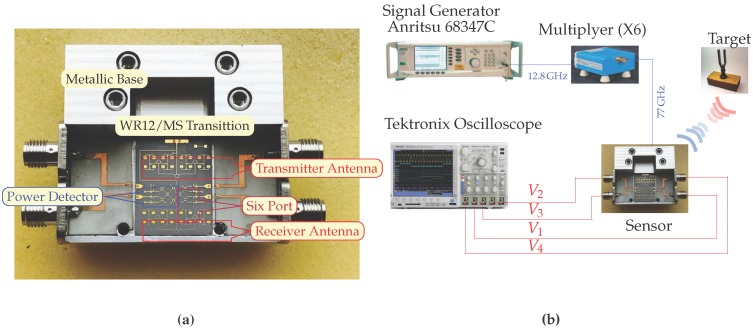
(**a**) Picture of the manufactured hardware prototype. (**b**) Short distance measurement setup.

Measurements are performed with our fabricated prototype. Its picture is shown in Figure 19a. The measurement setup includes the HP-83550 series mm-wave source, the mm-wave active multiplier from OML (X6) and the Tektronix DPO 7054 digital phosphor oscilloscope. The mm source frequency is set to 12.83 GHz and 10 dBm input power, then the multiplier will generate a 77-GHz signal with 0 dBm power at the input of the parallel line coupler. The die is mounted and power detectors are wire-bonded to the PCB using ribbon bonds to ensure good matching over a broad bandwidth. Preliminary experimental distance and frequency measurements were performed in our laboratory. In Figure 19b, one can observe the tuning fork experiment block diagram.

**Figure 20:**
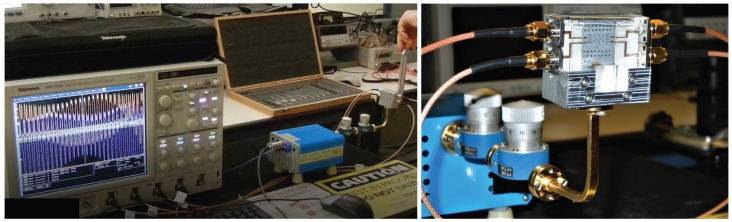
Picture of the tuning fork experiment setup.

The displayed images on the oscilloscope screen are captured in the initial part of the fork vibration showing also the fork frequency modulated by the inherent hand displacement. In the other measurement, the fork was mounted on a stand to avoid this secondary amplitude modulation due to hand movements. The experimental setup is developed according to the block diagram of Figure 19a, as shown in Figure 20. An additional WR-12 attenuator and phase shifter were used in line with the frequency multiplier output, to be able to reduce the transmitter power and to change the phase of the transmitted signal. These allow one to explore the I/Q complex plane, as in system simulations.

**Figure 21:**
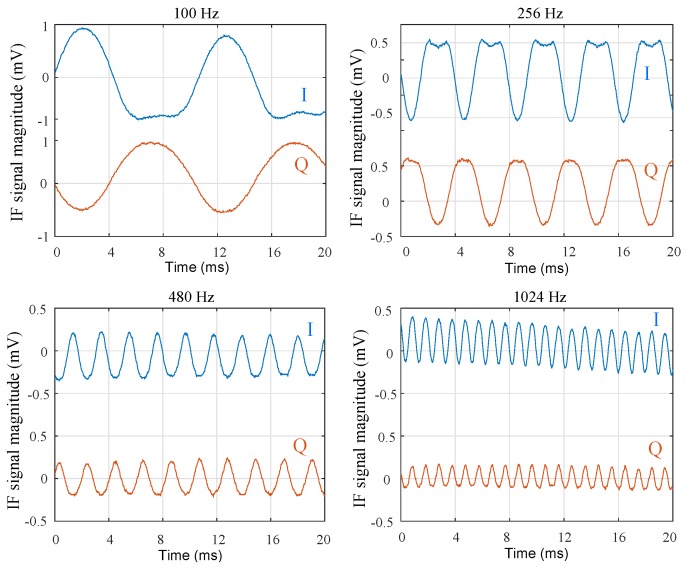
Measurement results for the four tuning forks at various frequencies (I in blue and quadrature (Q) in red).

The measurement results for I and Q are given in Figure 21. The results were extracted from the 20-ms screen data of the oscilloscope display. The screenshots were not taken exactly at the same time, and there were still distortions in the sinusoidal output signals. These distortions are due to the harmonics produced by the fork oscillations in the reflected signal, as can also be seen in the simulated results. For larger forks, having lower oscillation frequencies and larger oscillation movements, more harmonics were generated in the beginning, and therefore, distortions of the measured signal were observed on the oscilloscope screen. The measurement results clearly present the expected frequency values for different tuning forks. The measured frequency of the first harmonic, using the measurement frequency tool of the digital oscilloscope, was equal to the mechanical oscillation frequency of the calibrated tuning fork, with a ±1-Hz accuracy.

## 5. Conclusions

The design of a mm-wave CW short-range sensor is proposed. It is integrated using an MHMIC technology and mounted on a metallic fixture for laboratory measurements. It consists of a wide-band six-port interferometer and of two antenna arrays of 16 patch elements, which are used for the transmitter and the receiver. The interferometer has a wideband of 12 GHz, and it operates at 77 GHz in this work. Four high sensitivity power detectors are used to obtain quadrature differential signals. Each detector is fabricated using a pair of HSCH-9161 Schottky diodes with a hybrid coupler to improve matching over the entire operating band. The reflected signal from the target is compared in the interferometer to the reference signal obtained using a directional coupler from the mm-wave oscillator. The hardware design of the radar sensor and its implementation to obtain raw data without the need of six-port calibration is given. To the best of our knowledge, this is the first six-port interferometer designed at 77 GHz on a 127-μm ceramic substrate. It is also the first low-cost 77-GHz radar sensor designed on such a small substrate. The power consumption of the radar sensor is low, at around 0 dBm in transmission, and it requires a low LO power of less than −20 dBm for a six-port reference signal. An experiment using tuning forks was performed to show the frequency measurement accuracy of the proposed sensor. The measured frequency using the oscilloscope of both quadrature signals generated by the six-port interferometer is equal to the audio frequency of each fork, for all the tested forks, from 100 Hz–1024 Hz. Accurate measurement results in a 10 μm range for the six-port circuit, related to circuit phase errors, and in the 20 μm range are achieved for the entire prototype. The proposed sensor is simple, robust and low-cost, and it can be used in various industrial or biomedical applications.

References

## Figures and Tables

**Table 1:** Simulated return losses of the V-band power detector.

	72 GHz	74 GHz	76 GHz	78 GHz	80 GHz	82 GHz
$S_{11}$	−30.03	−33.69	−35.21	−35.62	−32.85	−30.19

## References

[1] Hasch J., Topak E., Schnabel R., Zwick T., Weigel R., Waldschmidt C. (2012). Millimetre-wave technology for automotive radar sensors in the 77 GHz frequency band. IEEE Trans. Microw. Theory Tech..

[2] Jahn M., Feger R., Wagner C., Tong Z., Stelzer A. (2012). A four-channel 94-GHz SiGe-based digital beamforming FMCW radar. IEEE Trans. Microw. Theory Tech..

[3] Kuhne R.D. From vision to reality mobile communication application for traffic control and traffic management. Proceedings of the Intelligent Transport System Telecommunication.

[4] Feger R., Pfeffer C., Scheiblhofer W., Schmid C.M., Lang M.J., Stelzer A. (2013). A 77-GHz Cooperative Radar System Based on Multi-Channel FMCW Stations for Local Positioning Applications. IEEE Trans. Microw. Theory Tech..

[5] Tatu S.O., Wu K. (2013). Communication and Sensing Applications of Six-Port Technology. Microw. Rev..

[6] Jaeschke T., Bredendiek C., Kuppers S., Pohl N. (2014). High-precision DBand FMCW-radar sensor based on a wideband SiGe-transceiver MMIC. IEEE Trans. Microw. Theory Tech..

[7] Pohl N., Gerding M., Will B., Musch T., Hausner J., Schiek B. (2007). High precision radar distance measurements in overmoded circular waveguides. IEEE Trans. Microw. Theory Tech..

[8] Pichler M., Stelzer A., Gulden P., Vossiek M. Influence of systematic frequency-sweep non-linearity on object distance estimation in FMCW/FSCW radar systems. Proceedings of the 33rd European Microwave Conference.

[9] Droitcour A.D., Boric-Lubecke O., Lubecke V., Lin J., Kovacs G. (2004). Range Correlation and I/Q performance benefits in single chip silicon Doppler radars for non-contact cardiopulmonary signs sensing. IEEE Trans. Microw. Theory Technol..

[10] Park B.K., Boric-Lubecke O., Lubecke V.M. (2007). Arctangent Demodulation With DC Offset Compensation in Quadrature Doppler Radar Receiver Systems. IEEE Trans. Microw. Theory Tech..

[11] Yavari E., Boric-Lubecke O. Low IF demodulation for physiological pulse Doppler radar. Proceedings of the IEEE MTT-S International Microwave Symposium (IMS).

[12] Tise B.L., Dubbert D.F. (2005). Digital intermediate frequency receiver module for use in airborne SAR applications. US Patents.

[13] Wang G., Gu C., Inoue T., Li C. (2013). A hybrid FMCW-interferometry radar for indoor precise positioning and versatile life activity monitoring. IEEE Trans. Microw. Theory Tech..

[14] Wang G., Gu C., Inoue T., Li C. Hybrid FMCW-interferometry radar system in the 5.8 GHz ISM band for indoor precise position and motion detection. Proceedings of the 2013 IEEE MTT-S International Microwave Symposium Digest (MTT).

[15] Moldovan E., Tatu S.O., Gaman T., Wu K., Bosisio R.G. (2004). A New 94 GHz Six Port Collision Avoidance Radar Sensor. IEEE Trans. Microw. Theory Tech..

[16] Gu C. (2016). Short-range noncontact sensors for healthcare and other emerging applications: A review. Sensors.

[17] Li C., Xiao Y., Lin J. (2006). Experiment and Spectral Analysis of a Low-Power Ka-Band Heartbeat Detector Measuring from Four Sides of a Human Body. IEEE Trans. Microw. Theory Tech..

[18] Li C., Lin J. Non-Contact Measurement of Periodic Movements by a 22–40 GHz Radar Sensor Using Non-linear Phase Modulation. Proceedings of the 2007 IEEE/MTT-S International Microwave Symposium.

[19] Woods G.S., Maskell D.L., Mahoney M.V. (1993). A high accuracy microwave ranging system for industrial applications. IEEE Trans. Instrum. Meas..

[20] Engen G.F. (1969). An Introduction to the Description and Evaluation of Microwave Systems Using Terminal Invariant Parameters.

[21] Ghannouchi F.M., Mohammadi A. (2009). The Six-Port Technique.

[22] Mallat N.K., Moldovan E., Wu K., Tatu S.O. (2009). Millimeter-wave ultra-wideband six-port receiver using cross-polarized antennas. EURASIP J. Wirel. Commun. Netw..

[23] De Souza Rolim A.L., Belfort M.T. Six-port Complex Permittivity Measurements. Proceedings of the European Microwave Conference.

[24] Wang M., Haddadi K., Glay D., Lasri T. Compact near-field microwave microscope based on the multi-port technique. Proceedings of the European Microwave Conference, EuMC.

[25] Hentschel T. (2005). The six-port as a Communications Receiver. IEEE Trans. Microw. Theory Tech..

[26] Bosisio R.G., Zhao Y.Y., Xu X.Y., Abielmona S., Moldovan E., Xu Y.S., Bozzi M., Tatu S.O., Nerguizian C., Frigon J.F. (2008). New-Wave Radio. IEEE Microw. Mag..

[27] Hannachi C., Tatu S.O. A new compact V-band six-port receiver for high data-rate wireless applications. Proceedings of the 2015 IEEE Topical Conference on Wireless Sensors and Sensor Networks (WiSNet).

[28] Moldovan E., Tatu S.O. Design and characterization of novel W-band wide-band couplers and six-port circuit. Proceedings of the European Microwave Conference (EuMC).

[29] Schiel J.C., Tatu S.O., Wu K., Bosisio G. Six-port direct digital receiver (SPDR) and standard direct receiver (SDR) results for QPSK modulation at high speeds. Proceedings of the IEEE MTT-S International Microwave Symposium Digest.

[30] Venot Y., Wiesbeck W. 76.5 GHz radar sensor for contact-free distance measurement with micrometer accuracy. Proceedings of the 2003 IEEE Sensors.

[31] Tatu S.O., Moldovan E., Wu K., Bosisio R.G., Denidni T. (2005). Ka-Band Analog Front-end for Software Defined Direct Conversion Receiver. IEEE Trans. Microw. Theory Tech..

[32] Hammou D., Djerafi T., Nedil M., Tatu S.O. (2016). Design Considerations for On-wafer Millimeter Wave Measurements on Thin Ceramic Substrate. IEEE Trans. Meas..

[33] Arab H., Akyel C., Tatu S.O. Wide-band Millimetre Wave Down-converter Based on Six-port Circuit for Radar and Sensing Applications. Proceedings of the 2017 XXXIInd General Assembly and Scientific Symposium of the International Union of Radio Science (URSI GASS).

[34] Arab H., Tatu S.O., Akyel C. Performance analysis for two different structures of 77 GHz six-port correlator. Proceedings of the 2016 17th International Symposium on Antenna Technology and Applied Electromagnetics (ANTEM).

[35] Arab H., Tatu S.O., Akyel C. Design and Characterization of a 77 GHz Six-Port Modulator for an Automobile Radar. Proceedings of the 2016 IEEE 84th Vehicular Technology Conference (VTC-Fall).

[36] Gonzalez G. (2008). Microwave Transistor Amplifiers.

[37] Hammou D., Nedil M., Kandil N., Moldovan E., Tatu S.O. (2013). V-band millimetre-wave micro-strip to rectangular waveguide transition. Microw. Opt. Technol. Lett..

[38] Hammou D., Moldovan E., Tatu S.O. Novel MHMIC Millimeter Wave Power Divider/Combiner. Proceedings of the IEEE Canadian Conference on Electrical and Computer Engineering (CCECE 2011), Conference proceedings.

[39] Vinci G., Lindner S., Barbon F., Mann S., Hofmann M., Duda A., Weigel R., Koelpin A. (2013). Six-port radar sensor for remote respiration rate and heartbeat vital-sign monitoring. IEEE Trans. Microw. Theory Tech..

[40] Vinci G., Lindner S., Mann S., Barbon F., Linz S., Weigel R., Koelpin A. Six-port microwave interferometer radar for mechanical vibration analysis. Proceedings of the 2013 European Radar Conference.

[41] Deban R., Boutayeb H., Wu K., Conan J. (2010). Deterministic approach for spatial diversity analysis of radar systems using near-field radar cross section of a metallic plate. IEEE Trans. Antennas Propag..

[42] Jun X., Bo Y., Xin L., Juan C. (2007). Theoretical model and optimization of a novel temperature sensor based on quartz tuning fork resonators. Phys. Scr..

